# Supplementary material for: The interactome of intact mitochondria by cross-linking mass spectrometry provides evidence for coexisting respiratory supercomplexes
Source: Mol Cell Proteomics. 2017 Dec 8;17(2):216–32. doi: 10.1074/mcp.RA117.000470 (PMC5795388; doi:10.1074/mcp.RA117.000470)
Supplement: Supplemental Data [file supp_RA117.000470_133922_0_supp_23978_fzffwf.zip › spectra_annotation/mito_spectra_annotation/383-1-4-1-7-1.pdf]

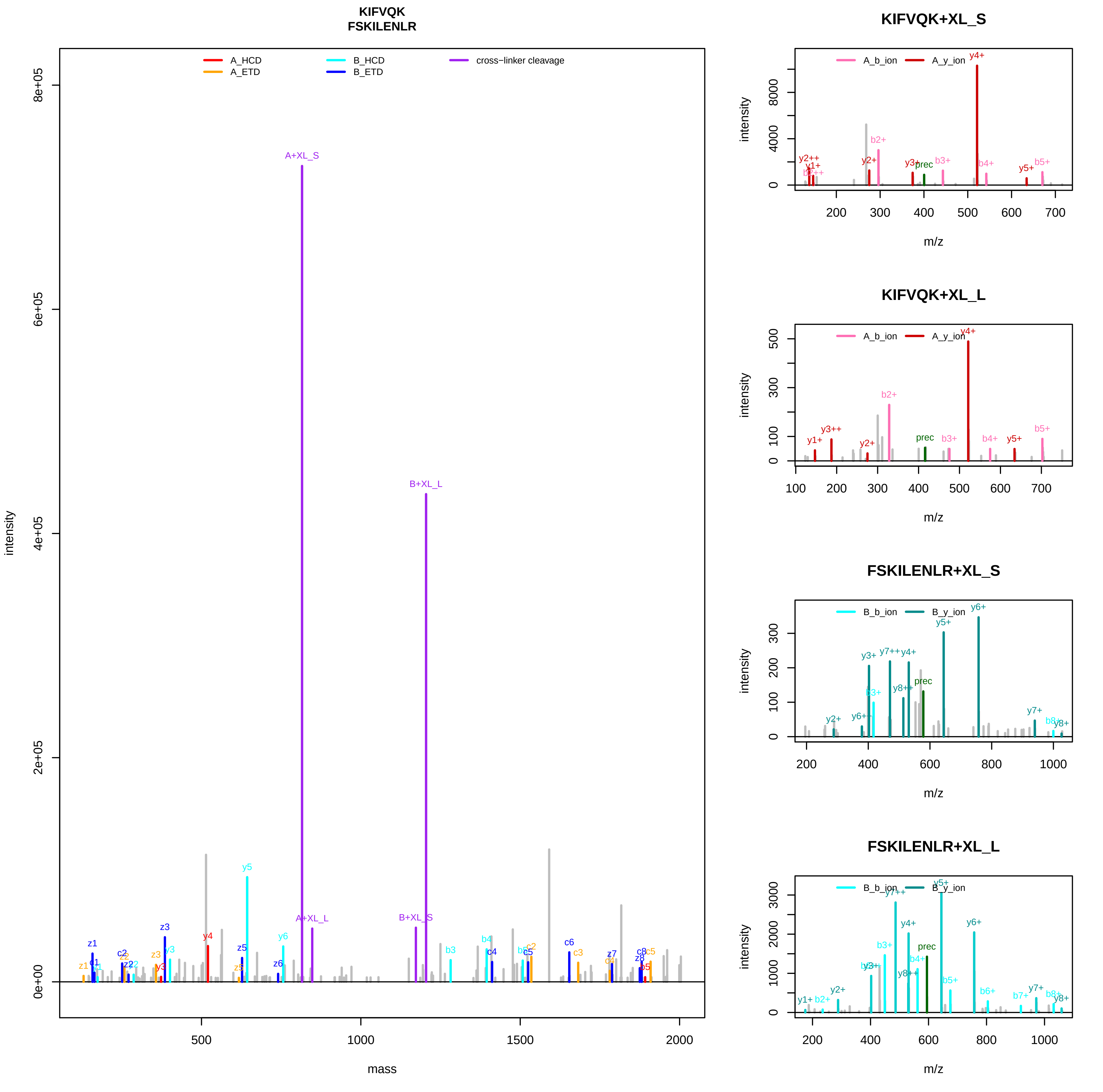
KIFVQK
FSKILENLR
A_HCD
A_ETD
B_HCD
B_ETD
cross−linker cleavage
A+XL_S
B+XL_L
A+XL_L
B+XL_S
y5
y6
y4
z3
z5
z6
z7
z8
c6
c3
c5
b3
b4
c4
intensity
mass
0e+00
2e+05
4e+05
6e+05
8e+05
500
1000
1500
2000
KIFVQK+XL_S
A_b_ion
A_y_ion
y4+
b2+
y2+
y3+
prec
b3+
b4+
y5+
b5+
m/z
KIFVQK+XL_L
y3++
y1+
FSKILENLR+XL_S
B_b_ion
B_y_ion
y6+
y5+
y3+
y7++
y4+
y8++
y7+
y8+
FSKILENLR+XL_L
b5+
b6+
b7+
b8+
